# Death by Fire

**Published:** 1897-05-15

**Authors:** 


### Death by Fire.

Of all forms of death that by fire is universally regarded as the most terrible, and undoubtedly death at the stake provided the ingenious torturers of the Middle Ages with the most horrible combination of agony and terror which even they could devise for the compassing of their ends. Yet those who lose their lives in conflagrations do not by any means always suffer physical pain. In many cases, no doubt, sharp terror is the one thing of which the victim is conscious, and in many more, strange as it may seem, consciousness plays no part, life ceasing painlessly and without a struggle.

The great factor in the deciding whether suffering shall be felt or not is the integrity of the organ by means of which suffering is felt; and thus it happens that the pain caused by physical injury depends more on the state of the brain than on the damage suffered by the body.

An example of this we see every day in our hospitals, where that very slight alteration in the quality of the blood supplied to the brain which can be produced by the inhalation of a little chloroform vapour suffices to render the patient absolutely unconscious of the severest torture, and may perchance leave him all the while lost in the happiest of dreams.

Now in great conflagrations gases are produced which have much the same effect, and it is a fact that of those who lose their lives in such catastrophes a considerable proportion pass into death without any evidence of having suffered. This result is produced especially when a fire has smouldered, when the access of air has at first been insufficient to cause complete combustion, and when that deadly gas, carbonic oxide, has sent its victims into lethal sleep before the actual flames have reached them.

Of those, however, who have evidently struggled and fought, and whose charred corpses are afterwards found in attitudes suggestive of violent efforts made in attempting to escape, it must not be imagined that they have of necessity been burned alive and have died in the agony which such contortions are popularly imagined to express.

Death from agony is really death from shock, a condition in which the body is limp and helpless; whereas in death from suffocation struggling may go on even after consciousness has passed, and the strained attitude of the corpse may be expressive only of the final paroxysmal effort made in a state of entire unconsciousness.

Suffocation in a fire depends on something more than mere carbonic acid poisoning. It is the stoppage of the breathing by the stifling vapours which does the mischief. Carbonic acid would doubtless kill if it could be breathed, but anyone who has attempted to enter a burning building will know that suffocation depends not on the stuff one breathes, but on the fact that one cannot breathe at all. The lungs are as much deprived of their supply of oxygen as if the sufferer were plunged over head in water, and the struggle produced is much the same.

While then we must admit the horror of the moment, the terror, the fight for breath, and finally the death from suffocation, we must remember that all this is often a matter of short duration, and that it is something very different from the slow torture of being burned alive.

The mental state in moments of great excitement is worthy of consideration in this connection. There is a good deal of evidence, gathered more especially from the experience of the battle field, tending to show that in times of great excitement the body is curiously insensible to pain. It would seem as if in such conditions, much as in the state of hypnotism, the consciousness were awake only to the subject in hand. The instances are so frequent in which more or less severe and painful wounds have only been discovered after the necessity for action had passed away that we are driven to hope and to believe that, so long as all the energies are absorbed in the effort to escape, actual suffering, even from fire, may not be great—may even be unfelt.

We wish we could stop here. But it must be recognised that while all this is true, there are cases in which people are actually burned alive—consumed little by little until the heart stops from shock.

Familiar pictures of martyrs at the stake show us one of the conditions for such an event; the wind driving the smoke aside so that the head, the brain, and thus the consciousness, are left intact until the heart is stopped by sheer agony.

It must always be remembered that no one can live or retain consciousness when actually within the flames. But those who are caught and held fast in the full blast of fresh air which is being drawn into the centre of the conflagration cannot be suffocated, they cannot make those violent efforts which would numb their consciousness of pain; they can but wait and suffer until, as the result of agony or terror, let us hope the latter, their hearts stand still. This is indeed a terrible fate.

